# From Protein Structure to Drug Discovery: Bioinformatics Breakthroughs in 2024–2025

**DOI:** 10.3390/cimb48010033

**Published:** 2025-12-26

**Authors:** Meiqi Fan, Quan Zou

**Affiliations:** 1 Yangtze Delta Region Institute (Quzhou), University of Electronic Science and Technology of China, Quzhou 324000, China; fanmeiqi93@foxmail.com; 2 Institute of Fundamental and Frontier Sciences, University of Electronic Science and Technology of China, Chengdu 610054, China

Bioinformatics is a key part of modern biomedical research that helps scientists understand complex biological systems with great accuracy [1,2,3]. This field combines computer science with molecular biology and medicine, and drives progress in disease research, drug development, and personalized healthcare [4,5,6]. As members of the Editorial Board for the “Bioinformatics and Systems Biology” section [7] of *Current Issues in Molecular Biology* (*CIMB*) (https://www.mdpi.com/journal/cimb, accessed on 19 December 2025), we are pleased to see the great progress made in this field during 2024–2025. Since it started, this section has published 157 high-quality papers and 12 Special Issues, covering a broad range of topics from computer modeling to clinical use, and showing that bioinformatics plays an important role in modern biomedical research.

Protein structure and function have always been important questions in biology, and great progress has been made in this area over the past two years [8,9,10,11]. The Special Issue “Structure and Function of Proteins: From Bioinformatics Insights” shows how computer methods help us understand the shape and movement of proteins, as well as how they function [12]. For instance, Ashley and colleagues wrote a detailed review of ADAR family proteins that helps us better understand RNA editing [13]. Their study combines computer methods with experiments to give us a deeper understanding of how protein structure, movement, and function are connected.

Moving from protein structure to drug discovery is an important use of bioinformatics, and the field of computer-based drug target prediction has grown a great deal thanks to progress in protein structure prediction and network biology [14,15,16,17,18]. The Special Issue “Predicting Drug Targets Using Bioinformatics Methods” presents progress in protein–protein interaction mapping, drug–protein interaction analysis, and RNA–/DNA–protein interaction modeling [19]. Yamkela and colleagues used computational analysis to show that SARS-CoV-2 non-structural proteins may interact with human heat shock proteins, providing new clues regarding viral replication [20]. Additionally, Xie and colleagues studied protein lactylation and proteomic features in cirrhosis patients treated with UC-MSC, and their work shows how bioinformatics supports precision medicine [21]. Studies like these speed up the growth of proteomics and pharmacomics and provide these fields with useful tools that make it possible to find and test drug targets.

Bioinformatics also helps researchers study complex diseases in new ways. The Collection “Bioinformatics and Systems Biology” shows the power of combining different computer tools, such as spatial transcriptomics, network pharmacology, and molecular docking [7]. Schmidt and colleagues made a spatial transcriptomics browser, that helps explore gene expression patterns in tissue environments [22]. Molecular network analysis is becoming more important in disease research and drug discovery, and Leblebici and colleagues studied how ovarian cells change into tumors using computer methods [23], providing the field with new ideas for targeted treatments. Singh and colleagues combined network pharmacology, molecular docking, and simulations to understand how cinnamon works in treating diabetic kidney disease [24]. These studies show the great potential of systems biology methods with regard to understanding complex diseases and finding treatment targets. Microbiome research has also gained from progress in bioinformatics, enabling Kirby and colleagues to use a gut microbiome model to test how nutrition affects gut bacteria structure and function [25]. This elucidates new views on the relationship between the microbiome and health.

Technological progress continues to guide the development of bioinformatics, and next-generation sequencing (NGS) remains a key technology in genomics and molecular biology [26,27,28]. The use of artificial intelligence in NGS is now a major trend, and Athanasopoulou and colleagues reviewed AI applications and challenges in NGS and noted that machine learning can improve the accuracy and speed of sequence analysis. In clinical settings, NGS-based liquid biopsy enables non-invasive detection of tumor-related nucleic acids, creating new possibilities for early cancer diagnosis and monitoring [29].

During 2024–2025, we have been witness to the fast development and deep integration of bioinformatics methods into the complete chain from protein structure to drug discovery. Looking ahead, new research themes show continued growth where genomics, systems biology, and computational medicine meet. We believe bioinformatics will continue to play revolutionary roles in our understanding of complex disease mechanisms, the exploration of targets, and the design personalized treatment plans.

The future of bioinformatics and systems biology has many opportunities. Consequently, CIMB is now accepting papers for several cutting-edge Special Issues, including “Linking Genomic Changes with Cancer in the NGS Era, 3rd Edition” [30], “Harnessing Genomic Data for Disease Understanding and Drug Discovery” [31], “Bioinformatics in Human Disease Network Analysis” [32], “Emerging Trends for Genome-Wide Association Studies in Complex Disease Genetics” [33], “Featured Papers in Bioinformatics and Systems Biology” [34], and “Emerging Trends for Genome-Wide Association Studies in Complex Disease Genetics” [35]. From protein structure prediction to drug discovery, from single-cell analysis to systems-level network modeling, bioinformatics methods are speeding up our understanding of living systems. They give strong support for precision medicine and personalized therapy. With the continued growth of AI technologies, the ongoing collection of multi-omics data, and big improvements in computing power, we expect to see even more groundbreaking progress in 2026 and beyond.

The “Bioinformatics and Systems Biology” section of CIMB is dedicated to publishing cutting-edge research in this field and welcomes researchers to submit innovative research findings on computational biology, systems modeling, machine learning applications, and bioinformatics applications in personalized medicine and drug discovery. Let us work together to advance the continuous development of bioinformatics and systems biology and contribute to addressing major challenges facing human health.
